# Singlet‐Oxygen Generation by Peroxidases and Peroxygenases for Chemoenzymatic Synthesis

**DOI:** 10.1002/cbic.202000326

**Published:** 2020-10-05

**Authors:** Kim N. Ingenbosch, Stephan Quint, Melanie Dyllick‐Brenzinger, Dennis S. Wunschik, Jan Kiebist, Philipp Süss, Ute Liebelt, Ralf Zuhse, Ulf Menyes, Katrin Scheibner, Christian Mayer, Klaus Opwis, Jochen S. Gutmann, Kerstin Hoffmann‐Jacobsen

**Affiliations:** ^1^ Niederrhein University of Applied Sciences Department of Chemistry and Institute for Coatings and Surface Chemistry Adlerstrasse 32 47798 Krefeld Germany; ^2^ Deutsches Textilforschungszentrum Nord-West gGmbH Adlerstrasse 1 47798 Krefeld Germany; ^3^ Institute of Physical Chemistry and CENIDE (Center for Nanointegration) University Duisburg–Essen Universitätsstraße 5 45117 Essen Germany; ^4^ Enzymicals AG Walther-Rathenau-Str. 49a 17489 Greifswald Germany; ^5^ Chiracon GmbH Im Biotechnologiepark 9 14943 Luckenwalde Germany; ^6^ Faculty of Environmental and Natural Sciences Brandenburg University of Technology Cottbus-Senftenberg Großenhainer Strasse 57 01968 Senftenberg Germany; ^7^ Present address: Leibniz Institute for Plasma Science and Technology Felix-Hausdorff-Strasse 2 17489 Greifswald Germany

**Keywords:** chemo-enzymatic synthesis, fluorescence, peroxidase, peroxygenase, singlet oxygen, singlet oxygen sensor green

## Abstract

Singlet oxygen is a reactive oxygen species undesired in living cells but a rare and valuable reagent in chemical synthesis. We present a fluorescence spectroscopic analysis of the singlet‐oxygen formation activity of commercial peroxidases and novel peroxygenases. Singlet‐oxygen sensor green (SOSG) is used as fluorogenic singlet oxygen trap. Establishing a kinetic model for the reaction cascade to the fluorescent SOSG endoperoxide permits a kinetic analysis of enzymatic singlet‐oxygen formation. All peroxidases and peroxygenases show singlet‐oxygen formation. No singlet oxygen activity could be found for any catalase under investigation. Substrate inhibition is observed for all reactive enzymes. The commercial dye‐decolorizing peroxidase industrially used for dairy bleaching shows the highest singlet‐oxygen activity and the lowest inhibition. This enzyme was immobilized on a textile carrier and successfully applied for a chemical synthesis. Here, ascaridole was synthesized via enzymatically produced singlet oxygen.

## Graphical abstract

**H_2_O_2_ to** ^**1**^ **O_2_ to…** The formation of singlet oxygen from hydrogen peroxide by heme peroxidases and peroxygenases, but not catalases, is shown by fluorescence spectroscopy. The most active peroxidase, based on kinetic analysis using the fluorescent trap singlet‐oxygen sensor green, was applied in a chemo‐enzymatic synthesis of ascaridole.

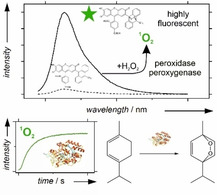

## Introduction

Reactive oxygen species (ROS) are involved in numerous metabolic activities, in intracellular signaling, regulating several kinases, transcriptional factors, in cytotoxicity and apoptosis.^[1]^ ROS include the nonradicals hydrogen peroxide (H_2_O_2_), organic hydroperoxides (ROOH), hypochlorous acid (HOCl) and the radical species of medium lifetime such as superoxide (O_2_^−^), the nitroxyl radical (NO^.^) as well as short‐lived diffusible entities such as hydroxyl (HO^.^), alkoxyl (RO^.^), peroxyl (ROO^.^), and singlet oxygen (^1^O_2_).^[2]^ Among the family of ROS, those exhibiting very high reactivities, including ^.^OH and ^1^O_2_, remain relatively underexplored. To combat ROS generated complications, cells have developed a complex enzymatic as well as a nonenzymatic antioxidant defense system in which peroxidases play an important role.^[3]^

Peroxidases are ubiquitous enzymes in all forms of life. In organisms, these enzymes serve not only the detoxification of reactive oxygen species but also the oxidation of numerous compounds by the use of ROS. For the oxidation process the co‐substrate binds to the active site, which contains either an iron protoporphyrin IX (heme peroxidases), other metals like vanadium, manganese, halogens (non‐heme peroxidases) or specific metal‐free prosthetic groups.^[4]^ Unspecific peroxygenases (UPO) are a relatively new class of enzymes, which belong to the peroxidase family but differ in the reaction mechanism of electron transfer.^[5]^

**Figure 1:**
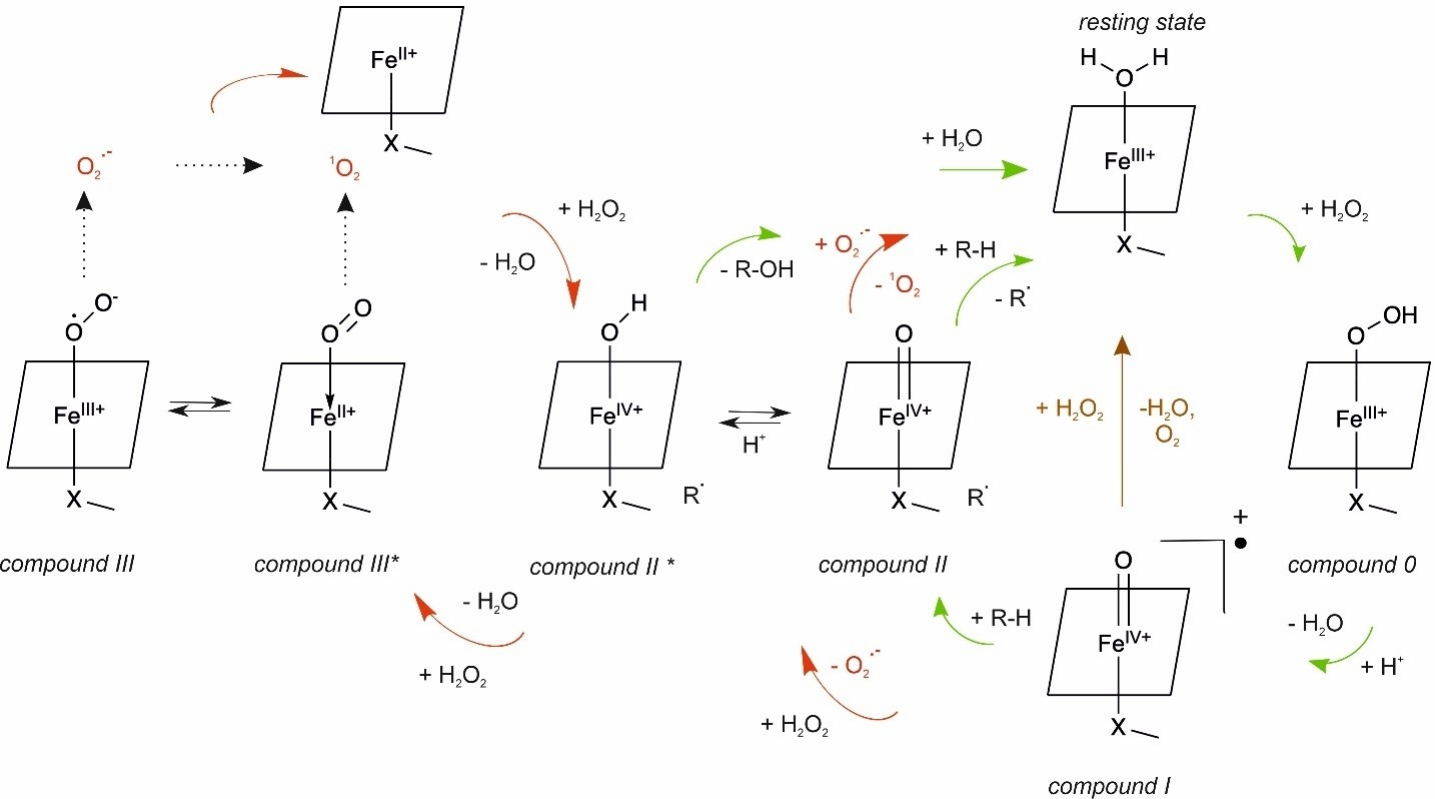
*Green arrows*: general mechanism for peroxidase and peroxygenase catalysis involving the substrates H_2_O_2_ and the organic compound R−H. The monooxygenase pathway of UPOs involving a two‐electron oxidation by oxygen transfer is typically illustrated with compound II*. The brown arrow indicates the catalase pathway. *Red arrows*: possible reaction paths to oxygen or ^1^O_2_.

Nowadays, chemists are becoming aware of the potential of peroxidases and peroxygenases in biocatalytic synthesis. Several peroxidases and peroxygenases are capable of activating inert or poorly activated C−H bonds and introducing oxygen functionalities into organic molecules by hydrogen peroxide.^[6]^ These oxyfunctionalization reactions are attractive green alternatives to transition metal catalysis.^[7]^ The catalytic mechanisms of peroxidases, peroxygenases and catalases involve a joint intermediate compound I (Figure 1). Subsequently, peroxidases release two radicals from two one‐electron oxidation steps, which undergo further coupling and disproportionation reactions. In contrast, peroxygenases can also perform direct oxygen transfer to the substrate in a two‐electron oxidation.[^8^, ^9^] In catalases, however, compound I decomposes to oxygen and water without the concurrent oxidation of a second substrate.

It is well known that peroxidases as horseradish peroxidase are capable of forming hydroxyl radicals in the presence of hydrogen peroxide under suitable reaction conditions. Here, a Fenton type mechanism has been suggested, which is facilitated by the formation of an iron(II) oxygen complex in excess of hydrogen peroxide.^[10]^ Recently, this reaction path has been detected for UPOs, too.^[11]^ Yet, the synthetic use of hydroxyl radicals is scarce.

Conversely, singlet oxygen is a valuable and green but rare chemical reagent that can oxidize electron‐rich organic compounds. Due to its low stability singlet oxygen has to be provided in situ. Typically, singlet oxygen is formed photocatalytically.^[12]^ The generation of singlet oxygen from sodium molybdate and hydrogen peroxide has also been described.^[13]^ Simply mixing hypochlorite with hydrogen peroxide will also produce ^1^O_2_.^[14]^ However, hypohalites can give rise to unwanted side reactions with organic substrates.

In the past decades, occasional reports of singlet‐oxygen formation by different enzymes have been released. Singlet oxygen production via hypochlorite formation by hydrogen peroxide/halide systems and chloroperoxidase, myeloperoxidase, lactoperoxidase, chloroperoxidase by chemoluminiscence^[15]^ has been reported earlier as well as the synthetic use of a vanadium chloroperoxidase.^[16]^ Direct singlet oxygen production from hydrogen peroxide by horseradish peroxidase has been detected occasionally by chemoluminescence as well as spectroscopically via the formation of a hydroperoxy‐dihydrofuran.^[17]^ Yet, no biocatalytic synthesis has been developed based on singlet oxygen production by iron peroxidases nor has a concise analysis of the enzymes capable of singlet‐oxygen formation been performed after the pioneering works from the 1980s.

The direct enzymatic production of singlet oxygen requiring only hydrogen peroxide in suitable amounts should be ideally suited for providing singlet oxygen for synthetic chemistry without the need of photochemical equipment or metal catalysis. The present study aims to determine the iron enzyme classes that are capable of forming singlet oxygen from hydrogen peroxide. The kinetics of singlet‐oxygen formation is elucidated in order to reveal relative activities and mechanistic differences of the enzymes under investigation. Different classes of heme proteins were analyzed: two heme peroxidases, two unspecific peroxygenases and three catalases were investigated in their ability to produce singlet oxygen by fluorescence spectroscopy. The commercial dye, singlet oxygen sensor green (SOSG), was used to trap singlet oxygen as a fluorescent SOSG endoperoxide. Finally, singlet oxygen is used in an exploratory chemical reaction, the synthesis of the natural terpene ascaridole.

## Results and Discussion

### Singlet‐oxygen formation by different hydrogen peroxide‐converting enzymes

**Figure 2:**
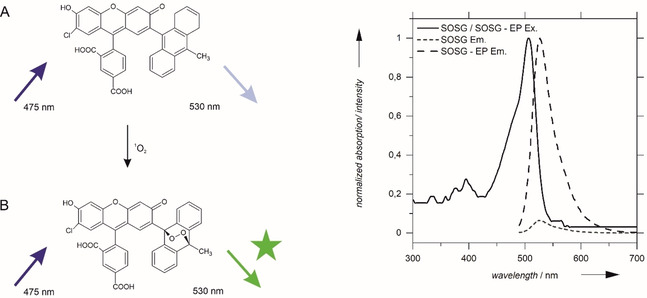
Left: Singlet oxygen trapping by SOSG leads to the formation of the fluorescent endoperoxide (SOSG‐EP). Right: Excitation and emission spectra of the SOSG dye and SOSG‐EP.

The singlet‐oxygen formation in the presence of hydrogen peroxide was analyzed by SOSG fluorescence. The SOSG endoperoxide is a fluorophore which can be excited at 475 nm at pH 6 and emits at 530 nm (Figure 2). SOSG is highly selective and can be used for the sensitive detection of singlet oxygen by fluorescence spectroscopy.^[18]^ The dye is used frequently in the detection and visualization of singlet‐oxygen formation in cellular environment.^[19]^ Yet, quantitative analyses of ^1^O_2_ formation have only been carried out very rarely,^[20]^ and a kinetic analysis is scarce.

In order to perform a quantitative analysis, the linearity of SOSG fluorescence intensity with singlet oxygen concentration was confirmed by photochemical synthesis of ^1^O_2_ in the presence of the photosensitizer Rose Bengal^[21]^ (Figure S1 in the Supporting Information). Hence, at the constant detector voltage and the constant SOSG concentrations applied for the following measurements, the SOSG‐EP fluorescence intensity is directly proportional to the concentration of ^1^O_2_. However, absolute ^1^O_2_ concentrations cannot be calculated due to the lack of an adequate calibration standard.

**Figure 3:**
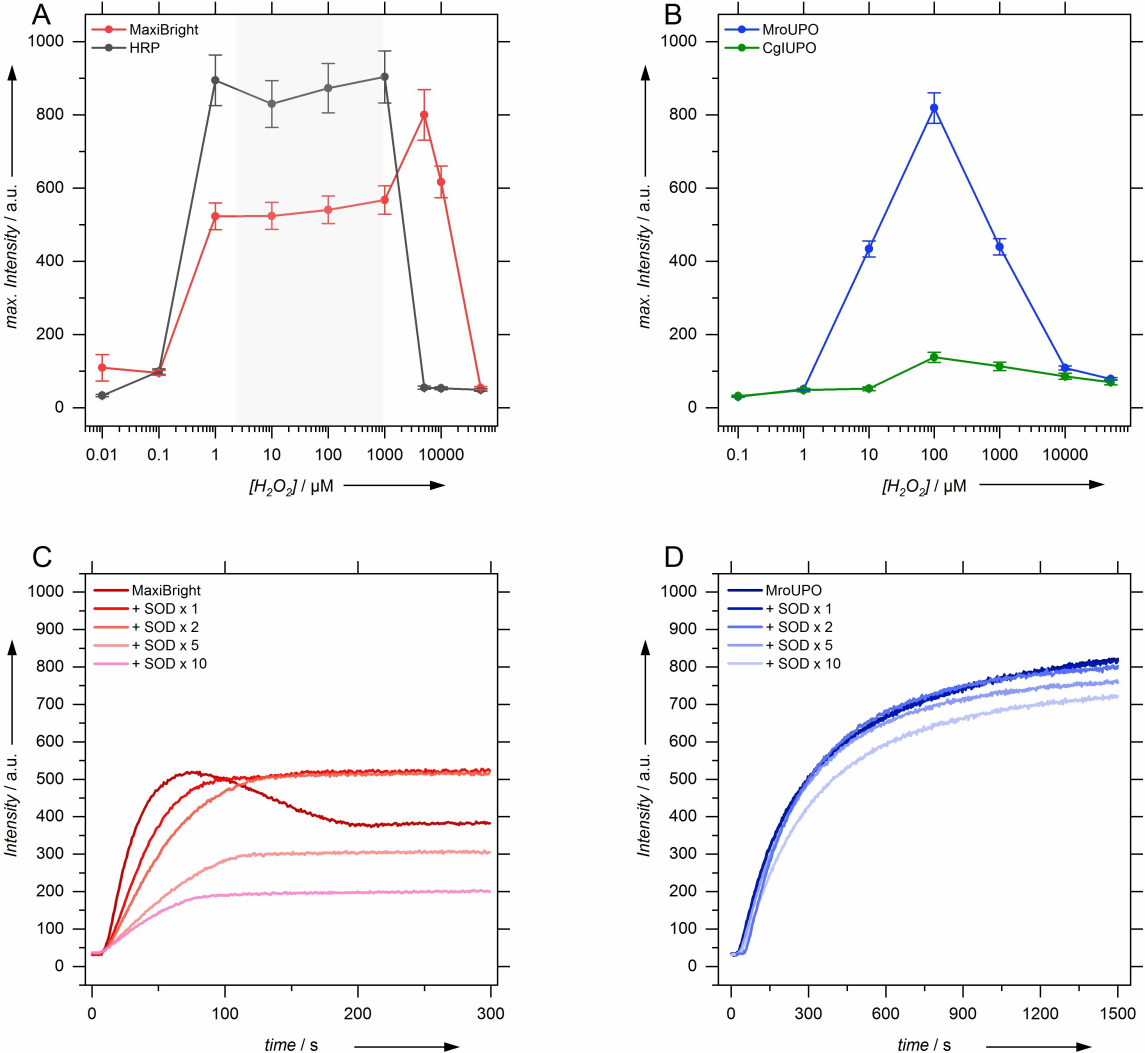
Top: Maximum fluorescence intensity of SOSG‐EP obtained from ^1^O_2_ production A) by the different peroxidases and B) by the peroxygenases at various hydrogen peroxide concentrations. The [H_2_O_2_] regime that is possibly affected by insufficient SOSG concentration for complete ^1^O_2_ capture is grayed out. Bottom: Example fluorescent time traces in the presence of increasing amounts of SOD for C) MaxiBright (1 μM H_2_O_2_) and D) *Mro*UPO (100 μM H_2_O_2_).

Singlet oxygen production by three different classes of enzymes accepting H_2_O_2_ as substrate are investigated: peroxidases, peroxygenases and catalases. Here, the commercial dye decolorizing peroxidase MaxiBright® (from *scorodonius*) and the horseradish peroxidase, type I (HRP), as well as the unspecific peroxygenases from *Marasmius rotula* (*Mro*UPO) and from *Chaetomium globosum (Cgl*UPO*)* were studied. The enzymatically produced singlet oxygen derived SOSG‐EP intensity is depicted in Figure 3 for the peroxidases and peroxygenases at various hydrogen peroxide concentrations. The figure shows that all peroxidases and peroxygenases under investigation are capable of forming singlet oxygen in the presence of hydrogen peroxide. Yet, *Cgl*UPO produces the lowest ^1^O_2_ concentrations. The catalases under investigation originate from *Aspergillus niger*, *Corynebacterium glutamicum* and *Micrococcus lysodeiticus*. Remarkably, no singlet‐oxygen formation was found with any catalase tested at any hydrogen peroxide concentration indicated.

Singlet‐oxygen formation via peroxidases and peroxygenases is detected in a broad range of hydrogen peroxide concentrations. This is challenging for a fluorescence spectroscopic technique, as a quantitative analysis of fluorescence spectroscopic data requires low dye concentrations in the low‐micromolar range in order to exclude inner filter and quenching effects. Hence, intensities in Figure 3 have to be analyzed with care as the maximum observable SOSG‐EP intensity is limited by the SOSG concentration. Thus, the plateau detected for the peroxidases is an artifact arising mainly from SOSG scarcity and too slow ^1^O_2_ capturing. (Figure S2) Yet, it may be concluded, that peroxidases show a broader substrate concentration regime and an earlier onset of singlet oxygen production than the peroxygenases.

As peroxidases are known to form superoxide in the presence of an excess of hydrogen peroxide,^[22]^ which might be the source of singlet oxygen,^[23]^ SOSG‐EP fluorescence was investigated in the presence of superoxide dismutase (SOD), a superoxide scavenger.^[24]^ As depicted in Figure 3C for MaxiBright, the SOSG‐EP fluorescence intensity decreases with increasing amount of SOD. The same effect was observed with HRP (data not shown). SOSG‐EP fluorescence is not quenched completely by a tenfold excess of SOD with respect to peroxidase. This shows that superoxide is a major source of singlet‐oxygen formation by the peroxidases but it is not the only source. Remarkably, SOD has only a minor effect on the SOSG‐EP fluorescence generated by the peroxygenases indicating a smaller fraction of superoxide related singlet‐oxygen formation (Figure 3D).

It is interesting to note that SOSG‐EP fluorescence time traces of the peroxidases show intensity maxima. This results from SOSG‐EP disintegration. The maxima vanish in the presence of SOD. No SOSG‐EP degradation is observed in the presence of peroxygenases under any condition tested. This emphasizes the role of superoxide formation by peroxidases which is also involved in a destructive reaction scheme of the SOSG‐EP chromophore.

### Analysis of the initial rate as observed by fluorescence spectroscopy

**Figure 4:**
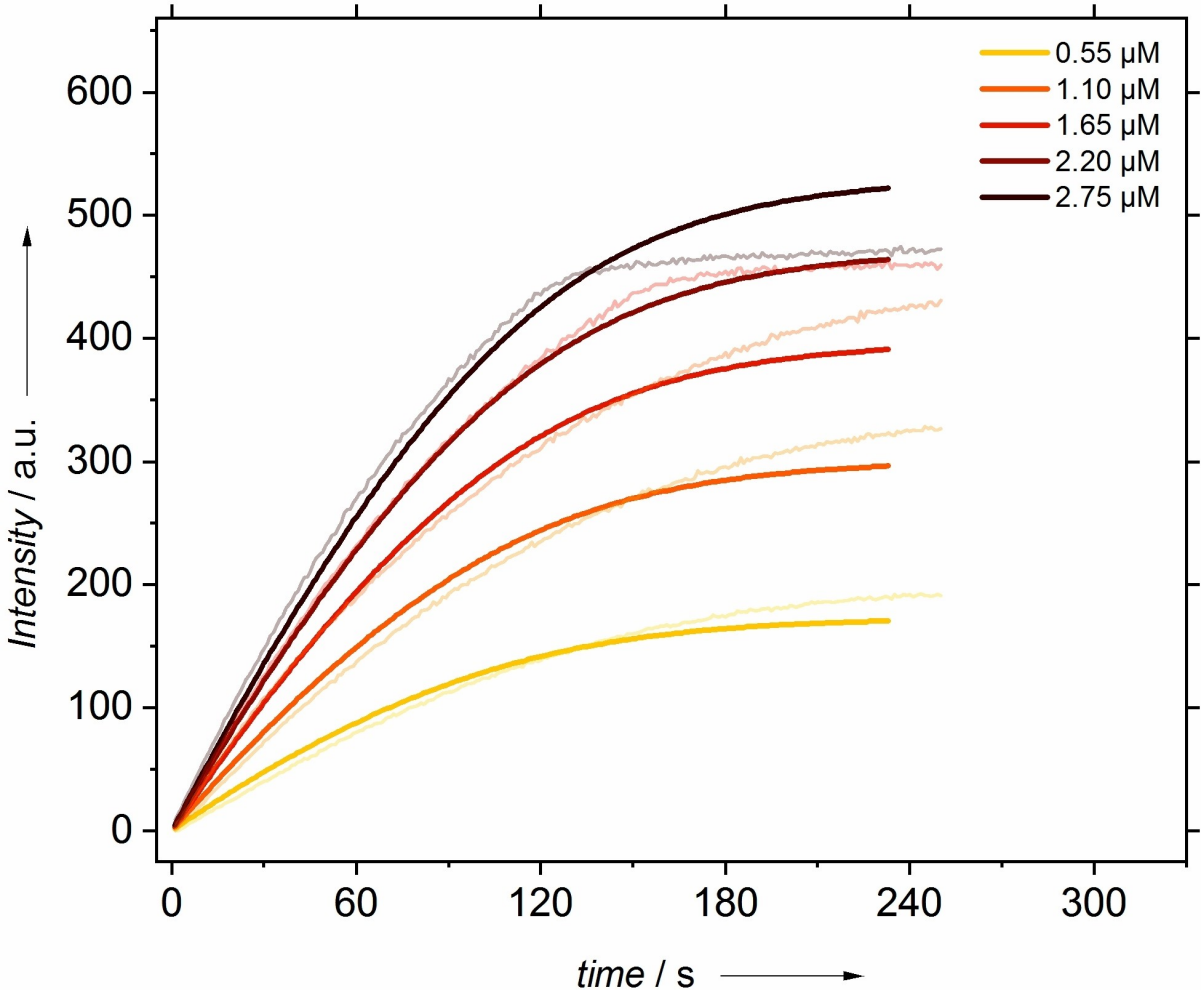
Kinetic time traces of the fluorescence intensity of SOSG‐EP in the presence of horseradish peroxidase and 1 μM H_2_O_2_. The SOSG concentration was varied over the indicated concentration range. The lines represent fits to the model presented in Figure 5.

**Figure 5:**
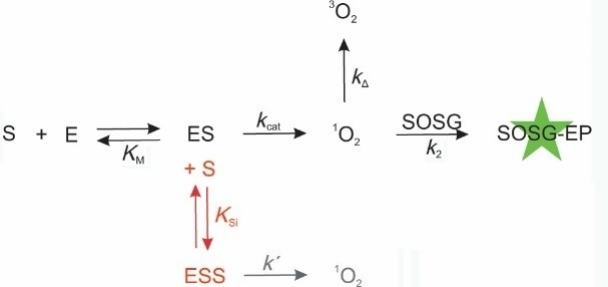
Kinetic model for the enzymatic reaction of the enzyme E with the substrate H_2_O_2_ (S) to singlet oxygen, including substrate inhibition (red), the spontaneous decay of singlet oxygen (*k*_Δ_), and the consecutive reaction with SOSG to the fluorescent endoperoxide SOSG‐EP.

The activity of the different enzymes for ^1^O_2_ formation was investigated by kinetic analysis. The initial rate of SOSG‐EP formation does not suffer from artificial intensity leveling as it is acquired at (initial) low product concentrations. However, Figure 4 reveals a dependency of the initial rate of SOSG‐EP formation on the SOSG concentration, which does not scale linearly with the dye content. This finding can be explained by incomplete ^1^O_2_ capture by SOSG. In competition to the reaction with SOSG, ^1^O_2_ can undergo spontaneous monomolecular decay to ^3^O_2_. We will show that this parallel reaction scheme (Figure 5) of a monomolecular decay and second order capture reaction leads to the observed dependency of SOSG‐EP formation on the SOSG concentration.

[SOSG]‐dependent kinetics were fitted to the numerical solution of the kinetic model with Matlab neglecting substrate inhibition at the low substrate concentration. With the lifetime of singlet oxygen of 3.1 μs determined by Egorov et al.^[25]^ and the Michaelis constant *K*_M_ acquired from a Michaelis–Menten fit to the first data points, fitting of the experimental data provided the bimolecular rate constant *k*_2_ for endoperoxide formation. Although the fit appears visually as moderate, *k*_2_ is a very robust fit parameter relying only on the *ratio* of the fluorescence intensities of the time traces at different SOSG concentrations. Hence, the successful fit verifies the suggested mechanism.

Enzymatic catalysis is typically described by Michaelis‐Menten kinetics. In this model, the dependency of the initial rate of catalysis on the substrate concentration [S] is described by Equation 1:

(1) $$v=v{}_{\max}\times \frac{\left[S\right]}{K{}_{M}+\left[S\right]}$$

with *v*_max_ being the maximum rate and *K*_M_ the Michaelis constant.

By application of the steady state approximation the rate law for SOSG‐EP formation is given by (Supporting Information):

(2) $$\frac{d[\mathrm{SOSG}\text{-}\mathrm{EP}]}{dt}=\frac{k{}_{2}\;\left[\mathrm{SOSG}\right]}{k{}_{\Delta }+k{}_{2}\;\left[\mathrm{SOSG}\right]}\times \frac{k{}_{\mathrm{cat}}\;\left[E\right]\left[S\right]}{K{}_{M}+\left[S\right]}$$

with E being the enzyme and S hydrogen peroxide. Equation (2) shows that Michaelis‐Menten type kinetics are observed at a constant SOSG concentration, where *k*_cat_ is modified by the dimensionless factor *f*.

(3) $$f=\frac{k{}_{2}\;\left[\mathrm{SOSG}\right]}{k{}_{\Delta }+k{}_{2}\;\left[\mathrm{SOSG}\right]}$$

Hence, a Michaelis‐Menten analysis is feasible and gives correct Michaelis constants. Values of *k*_cat_ differ from the real value by a constant factor (Table 1) and can thus be compared quantitatively with each other.

**Table 1:** Parameters of the fit to the [SOSG] dependent time traces given in Figure 4. K_M_ and k_Δ_ are input values of this fit. For the definition of the kinetic constants, see Figure 5.

*K*_M_ [μM]	*k*_Δ_ [s^−1^]	*k*_2_ [μM^−1^ s^−1^]	*f*
0.24±0.13	32 000	15 000±300	0.02

### Michaelis‐Menten kinetics of singlet‐oxygen formation

**Figure 6:**
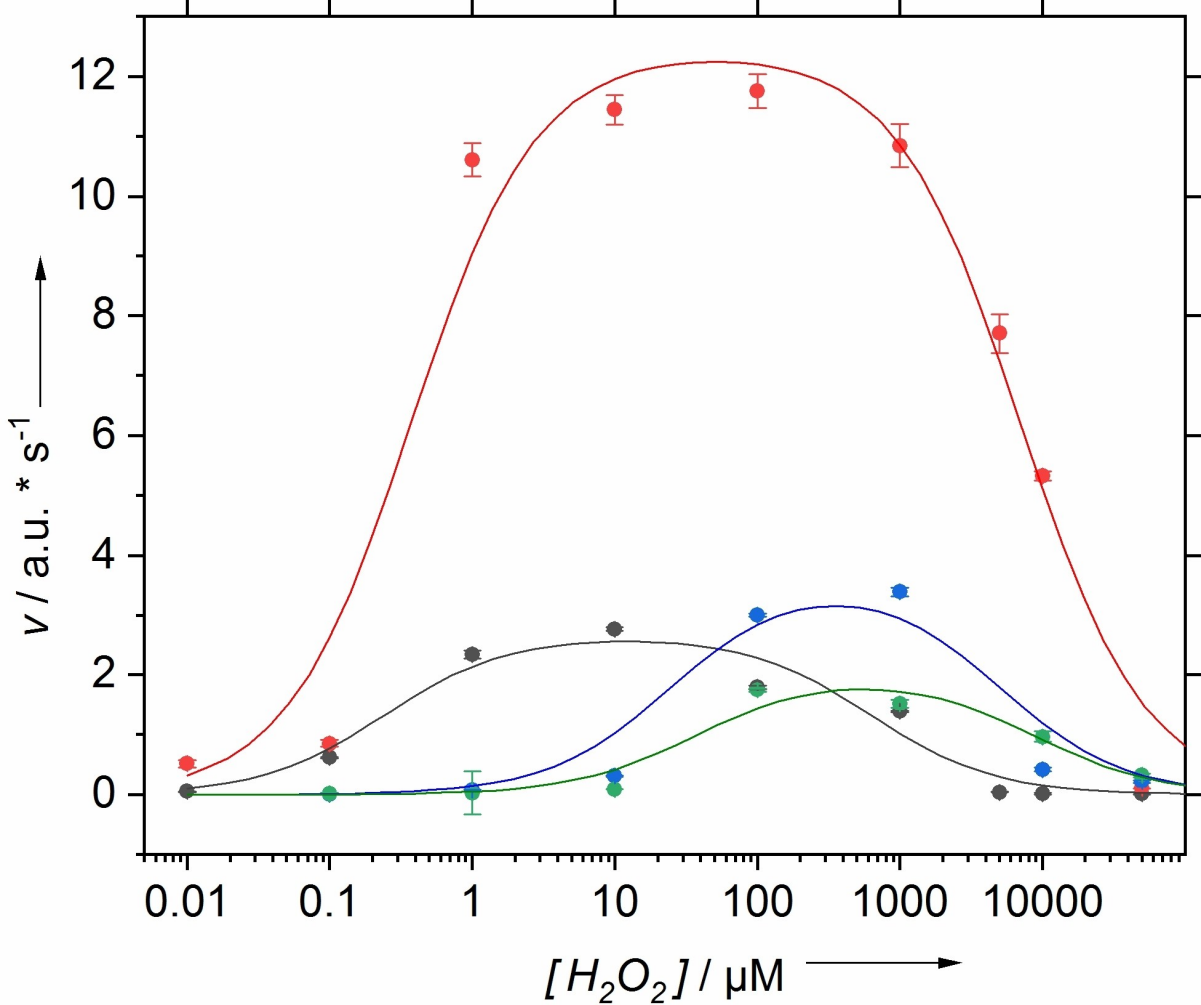
Michaelis–Menten plots of the initial reaction rate versus substrate concentration for the peroxygenases *Mro*UPO (blue) and *Cgl*UPO (green) and the peroxidases HRP (gray), MaxiBright (red). The lines represent the fits to Equation (4).

The initial rate of SOSG‐EP formation was determined over a broad range of hydrogen peroxide concentrations for the peroxidases and peroxygenases under investigation (Figure 6). According to Michaelis‐Menten kinetics, saturation behavior is expected. A qualitative assessment shows a strong deviation from Michaelis‐Menten kinetics for all enzymes under investigation. Accordingly, the respective linearization plots fail to give classical Michaelis–Menten parameters. In contrast, both peroxidases show broad maxima in reaction rate, which indicate substrate inhibition.

It is well known that hydrogen peroxide is a substrate with a strong inhibitory effect on numerous enzymes as peroxygenases and peroxidases. The model by Yoshino et al.^[26]^ for uncompetitive substrate inhibition was used for kinetic data analysis. Here, substrate inhibition is displayed by the binding of an additional substrate to a non‐catalytic site, which is characterized by the dissociation constant *K*_Si_ leading to a reduced catalytic rate constant *k′* in the model (Figure 5). The initial rate is described by Equation 4.

(4) $$v=v_{max}\cdot \frac{\left(1+\frac{\left[S\right]}{K_{Si}}\cdot \frac{k{}^{'}}{k_{cat}}\right)\left[S\right]}{K_{M}+\left[S\right]\left(1+\frac{\left[S\right]}{K_{Si}}\right)}$$

*K*_Si_ is the dissociation constant of the inhibited enzyme substrate complex, *k′* depicts the respective rate constant and *k*_cat_ the rate constant of the native enzyme substrate complex.

First, *K*_M_ and the apparent *v*_max_ were determined. All enzymes show Michaelis‐Menten behavior in a limited regime at low substrate concentrations which varies from enzyme to enzyme. Michaelis‐Menten parameters were estimated from the substrate concentration [S] dependent reaction rate *v* data by fits to Equation (1) in this regime. These were used as initial parameters for the fit of the experimental substrate dependency of the initial rate to Equation (4). The Michaelis constants (Table 2) correspond with previous kinetic studies of peroxidases.^[27]^ The analysis reveals that MaxiBright shows the highest *k*_cat_ of the peroxidases. Peroxygenases have a remarkably lower singlet‐oxygen formation activity represented in higher *K*_M_ values.

**Table 2:** Michaelis‐Menten parameters *K*_M_ for singlet‐oxygen formation, apparent *v*_max_ and *k*_cat_ for SOSG‐EP formation and the inhibition constant *K*_Si_.

Enzyme	*K*_M_ [μM]	*v*_max_ /Int. [a.u.][s^−1^]	*K*_Si_ [μM]	*k*_cat_ /Int. [a.u.][s^−1^ μM^−1^]
HRP	0.24±0.13	2.66±0.27	620±300	13±1.3
MaxiBright	0.37±0.16	12.42±0.81	7000±2100	83±5.5
*Mro*UPO	25±20	3.61±0.39	5000±3800	21±2.3
*Cgl*UPO	38±27	1.99±0.7	8600±5300	7.0±1.3

The inhibition model allows describing the Michaelis‐Menten plots of singlet oxygen production by peroxygenases and peroxidases as illustrated in Figure 6. This gives strong evidence that substantial substrate inhibition by hydrogen peroxide occurs. Interestingly, the kinetic constant of the hydrogen peroxide bound enzyme substrate complex *k′* was found to approach zero for all enzymes. This represents complete inhibition upon binding of additional hydrogen peroxide and the model simplifies to

(5) $$v=v_{max}\cdot \frac{\left[S\right]}{K_{M}+\left[S\right]\left(1+\frac{\left[S\right]}{K_{Si}}\right)}$$

The different enzymes vary in the response to hydrogen peroxide as described by the dissociation constantKSi . The analysis shows that MaxiBright features the highest reaction rate and stability.

### Enzymatically produced singlet oxygen as chemical reagent

**Figure 7:**
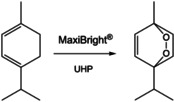
Conversion of α‐terpinene to ascaridole by hydrogen peroxide (urea‐H_2_O_2_: UHP) and the peroxidase MaxiBright.

Singlet oxygen is a valuable and green but rare chemical reagent. In an exploratory reaction, we performed the synthesis of the natural terpene ascaridole as depicted in Figure 7. Ascaridole is used as flavoring and as an antiparasitic agent. The synthesis of ascaridole is performed by the [4+2] cycloaddition of singlet oxygen to α‐terpinene.

The peroxidase MaxiBright was immobilized on a textile carrier as described recently for improved stability in organic solvent.^[28]^ Ethanol, acetonitrile and THF buffer mixtures were tested as solvents showing highest product yields in a buffer/acetonitrile mixture (1 : 10). The pH was varied in the range from 4 to 6. Keeping the pH below 5 achieves highest yields. The introduction of hydrogen peroxide via slow release from urea hydrogen peroxide proved to be a key step in increasing yields to a maximum of 13 %. Thus, a constant but low concentration of hydrogen peroxide is maintained. Moreover, urea hydrogen peroxide shows a higher solubility in organic media. Reaction times were varied from one to five days, with one day giving the highest yield of ascaridole. No reaction was observed in the absence of the enzyme.

**Figure 8:**
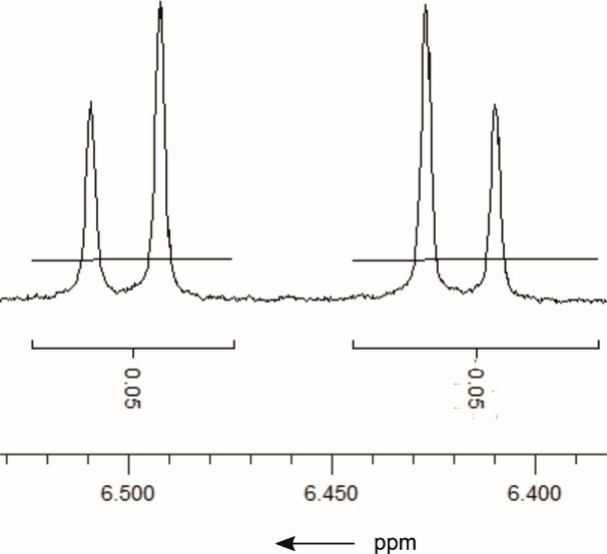
Section of the ^1^H NMR (400 MHz, CDCl_3_) spectrum of the crude product showing the doublets of the double bond of ascaridole at 6.50 ppm (1H, *J*: 8.5 Hz) and 6.40 ppm (1H, *J*: 8.4–8.6 Hz).

The crude product was analyzed by ^1^H NMR spectroscopy (Figure S3). The comparative ^1^H NMR data were taken from the literature,^[29]^ as well as supported by chemical synthesis of ascaridole. The signal that is assigned to the hydrogen atoms of the double bond of ascaridole can be clearly identified. The respective section is given in Figure 8. The depicted coupling pattern coincides with the values given in the literature for chemically synthesized ascaridole.^[29]^ The conversion of α‐terpinene is 100 % as judged by NMR spectroscopy. The major side product (29 %) was identified as cymene. It is readily formed by elimination and subsequent aromatization.

No product formation could be observed with any catalase under investigation. Moreover, the vanadium chloroperoxidase from *Curvularia inaequalis*, which is known to form singlet oxygen via the hypochlorite pathway,^[30]^ did not give comparable product yields (ca. 1 %) using bromide as cocatalyst.

### Mechanism of singlet‐oxygen formation

The peroxidase mechanism involves the intermediates compound 0, I and II that are depicted in Figure 1. Excess of hydrogen peroxide leads additionally to the formation of compound III, which is an off‐pathway intermediate.[^31^, ^32^] These species give unique optical absorption spectra of the prosthetic group and can be identified by the Soret band (ca. 400 nm) and the position of the Q_00_ and Q_0v_ band in the region from 500 to 700 nm.^[33]^ HRP has been characterized in detail.^[34]^ Therefor the analysis will be performed with this peroxidase.

**Figure 9:**
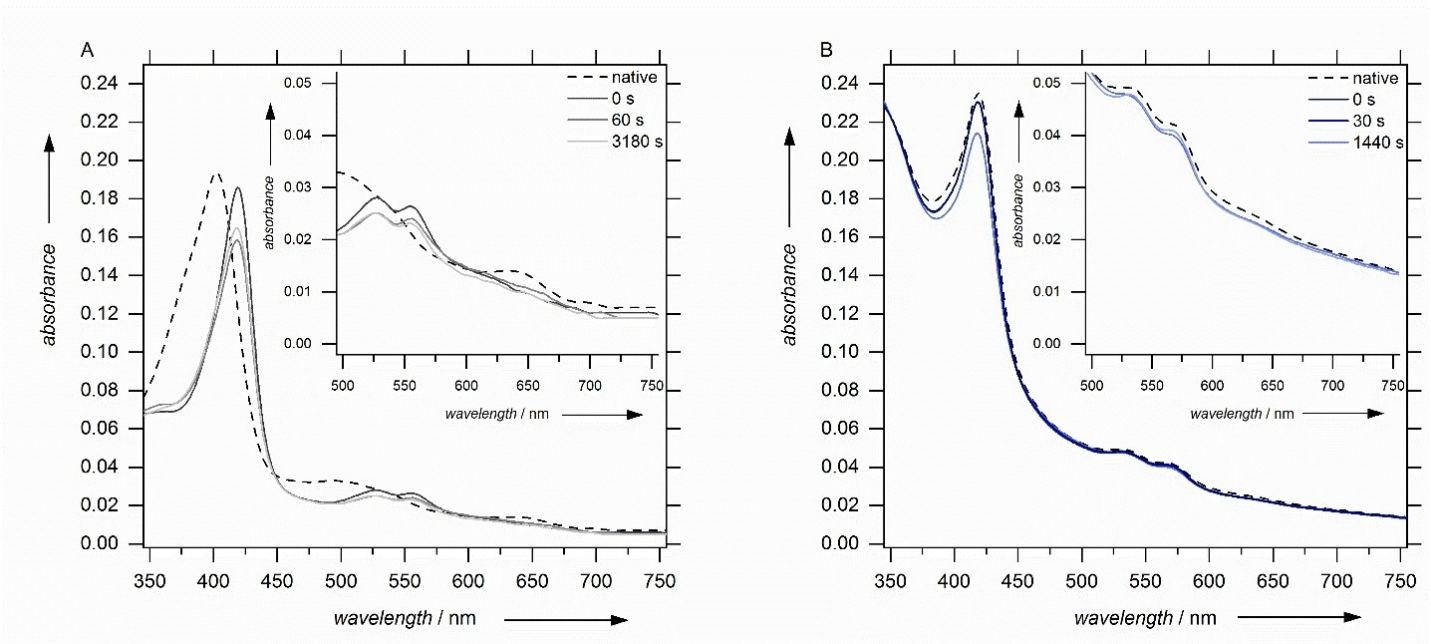
Transient optical absorption spectra of A) HRP upon addition of 10 μM hydrogen peroxide and B) *Mro*UPO upon addition of 1 mM hydrogen peroxide. 0 s refers to the immediate measurement after manual injection.

The absorption spectra of the peroxidase HRP under assay conditions showing maximum singlet‐oxygen formation rates are shown in Figure 9 (left). Immediately after hydrogen peroxide injection, changes in both regions are observed. The new absorption maxima at 419 nm and at 527 and 555 nm reflect compound II formation. The hydrogen peroxide concentration, where singlet oxygen production starts, coincides with the onset of new spectral features in the absorption spectra. At mM H_2_O_2_ concentrations compound III is identified by maxima at 543 and 577 nm (Figure S4). Moreover, the inhibited species P670 is found in the concentration region where inhibition is observed in the kinetics. Similar spectra were obtained from MaxiBright (data not shown).

In contrast, the absorption spectra of the singlet oxygen producing *Mro*UPO do not reveal new bands upon hydrogen consumption (Figure 9, right) but partial bleaching at any concentration tested (Figure S4). Yet, it has been shown previously that the absorption spectra of *Mro*UPO species are less specific than the spectra of HRP.^[11]^

## Discussion

Analysis shows that the peroxidases and *MroUPO* peroxygenase form singlet oxygen without hypochlorite intermediates from hydrogen peroxide at least in micromolar concentrations and on the timescale of minutes. In particular, the peroxidases accept hydrogen peroxide as oxidation and reduction substrate over a broad concentration range. This is reflected by inhibitory constants in the millimolar range. The successful synthesis of ascaridole by MaxiBright emphasizes the synthetic amounts of singlet oxygen produced even in the presence of organic solvents. The direct formation of singlet oxygen from hydrogen peroxide by peroxidases has only rarely been mentioned over the decades in literature and the synthetic potential of enzymatically produced singlet oxygen remains to be raised. In this exploratory study, α‐terpinene was chosen as a substrate because it produces a stable endoperoxide (ascaridole). Generally, a variety of conjugates dienes are potential substrates for this enzymatically induced [2+4] cycloaddition.

The lacking singlet‐oxygen formation by catalases is first surprising as oxygen formation by peroxidases and peroxygenase could be considered a catalase function. This shows that it is not compound I that is able to form singlet oxygen but compound II is involved. This idea is supported by our absorption spectroscopy analysis and the sequential H abstraction mechanism of catalases from hydrogen peroxide to oxygen.^[35]^ It has already been shown for myeloperoxidases that hydrogen peroxide cannot mediate the direct reduction of compound I of peroxidases to the resting state but mediates the conversion to compound II.^[36]^

In the absence of a reducing substrate peroxidases and peroxygenases react with hydrogen peroxide in a complex set of reactions to compound II of the regular peroxidase cycle.[^37^, ^38^, ^39^] In the peroxidase cycle, compound II formation includes the release of a substrate radical (Figure 1). In the absence of substrate, superoxide radical formation by peroxidases was found.^[38]^

Superoxide can be oxidized to singlet oxygen.^[40]^ It has been suggested previously that this superoxide decay can promote catalytic activities involving singlet oxygen.^[41]^ Yet, the yield of singlet oxygen from superoxide is marginal (0.2 %) in solution.^[42]^ A reaction with ferric peroxidase to oxygen and the resting state might increase the yield.^[43]^ Thus, the catalytic cycle is closed. This path represents one possible origin of singlet‐oxygen formation by peroxidases (Figure 1, red arrows). The suggested mechanism is in line with the observed dependence of the reaction rate of peroxidases on SOD concentration. Moreover, the low onset of hydrogen peroxide concentration required for ^1^O_2_ formation by peroxidases and their optical absorption spectra in the micromolar range support the idea of compound II being the reactive species.

Under further excess of hydrogen peroxide, peroxidases are known to form compound III, an oxyheme species.^[37]^ This corresponds to the optical absorption spectra at the higher hydrogen peroxide concentrations probed herein. Compound III has been identified as the source of catalytic activities of peroxidases that are independent of the peroxidase cycle.[^11^, ^41^] The nature of Fe−O_2_ bonding in oxyheme has been a subject of active debate for decades. Pauling assigned a singlet state to dioxygen,^[44]^ Weiss proposed a ferric‐superoxide complex,^[45]^ and McClure, Goddard and Olafson suggested the “ozone” model.^[46]^ Theoretical investigations as well as spectroscopic analysis indicate that oxyheme is multiconfigurational and comprises singlet oxygen as minor species.[^47^, ^48^] We suggest that compound III is the second source of singlet oxygen which might be either formed directly from Fe‐^1^O_2_ or indirectly from superoxide (Figure 1, left side).[^31^, ^48^] In contrast to the first path, this mechanism should not be disrupted by superoxide removal. The singlet oxygen production by *Mro*UPO which only starts at high hydrogen peroxide concentrations and is less sensitive to SOD rather accords with this second mechanism. The partition between both mechanisms is supposed to depend on the hydrogen peroxide concentration and the enzyme type.

It has been reported that hydroxyl radicals are also formed by peroxygenases and peroxidases in the absence of electron donor substrate.[^10^, ^11^] Concomitantly, a “catalase malfunction” was suggested involving the production of OH radicals by the Haber‐Weiss reaction of compound III.^[11]^ This supports the idea that peroxygenases form compound III in the absence of a second electron donor substrate. The present data indicate, that singlet oxygen is formed predominantly by compound III at intermediate hydrogen peroxide concentrations (μM‐1 mM regime) whereas OH radicals are formed at higher concentrations (>1 mM regime).

The formation of different ROS in the reaction is supposed to be a major obstacle in the synthetic application of the presented enzymatic method as this will lead to side reactions. Future work should concentrate on the elucidation of the detailed mechanism and the means to control the reaction path.

## Conclusion

In this study, we prove that peroxidases and peroxygenases are capable of forming synthetic concentrations of singlet oxygen in the presence of hydrogen peroxide. We can clearly exclude catalases from sharing this characteristic activity. The fluorescent trap SOSG allows a kinetic analysis after appropriate modeling of the reaction cascade leading to the fluorescent SOSG endoperoxide, revealing uncompetitive substrate inhibition. The commercial dye decolorizing peroxidase MaxiBright can be identified as the most active singlet oxygen‐forming enzyme which is the least prone to substrate inhibition. The successful synthesis of ascaridole by MaxiBright catalysis shows that this enzyme can be used in chemo‐enzymatic synthesis involving singlet oxygen as reagent.

## Experimental Section

**Materials**: The commercially available dye decolorizing peroxidase MsP1 from *M. scorodonius* marketed as MaxiBright® and produced by *A. niger* was a kind gift from DSM (Food Specialties B.V., Delft, Netherlands). Horseradish peroxidase type 1 was purchased from Sigma–Aldrich as lyophilized powder. The UPOs from the fungi *M. rotula* (*Mro*UPO) and *Chaetomium globusom* (*Cgl*UPO) were isolated as described previously.^[49]^ All enzymes were prepared as 1 % stock solutions in 50 mM sodium phosphate buffer pH 6 before use. SOSG was acquired from ThermoFisher. Vanadium chloroperoxidase from *C. inaequalis* (*Ci*VHPO) was expressed in *Escherichia coli*.^[50]^ Manganese superoxide dismutase from *E.coli* (Sigma) was a kind gift from Prof. Katja Ferenz (University Hospital, Essen). All chemicals were purchased from Roth (Germany).

**Singlet oxygen assay**: 100 μg of SOSG was freshly diluted in 1 mL 3 % methanol in phosphate buffer solution, handled on ice and discarded after each day. This dye stock solution was diluted in 50 mM degassed phosphate buffer (pH 6) before measurement. The final dye concentration used during the assay was 0.55 μM. The residual solvent content was 0.01 % and the residual oxygen concentration was below 1 mg/L. The temperature was 20 °C. Hydrogen peroxide dilutions were freshly prepared before analysis from a 30 % stock solution. The final enzyme concentration used in SO assay was 0.01 g/L. This corresponds to [HRP]=0.23 μM; [MaxiBright]=0.15 μM, [*Mro*UPO]=0.17 μM and [*Cgl*UPO]=0.28 μM.

Fluorescence emission of the SOSG dye was measured with a Varian Cary Eclipse Fluorescence Spectrometer (*λ*_ex_=475 nm, *λ*_em_=530 nm) providing a time resolution of 50 ms at the beginning of the time traces. The detector voltage was 700 V for all data except the dye dependency. Here, it was 550 V for the variation of [SOSG] at 1 μM H_2_O_2_ and 600 V for the combined variation of substrate and [SOSG].

^1^O_2_ formation in the presence of SOD was analyzed at 1 μM hydrogen peroxide concentration. Peroxidase and peroxygenase concentration was 0.01 g/L, pre‐mixed with 0.55 μM SOSG and SOD, reaction was started with the injection of H_2_O_2_. SOD was introduced with different mass equivalents with respect to the peroxidase/peroxygenase.

**Ascaridole synthesis**: 10 mL acetonitrile were mixed with 1 mL citrate phosphate buffer, pH 5 (ca. 0.1 M). 0.39 mL α‐terpene (2.4 mmol) were added. 1 cm^2^ textile carrying MaxiBright was cut into four pieces and added. The mixture was stirred for 10 min before 2 g urea/H_2_O_2_ (9.2 equiv.) were added. The reaction mixture was stirred overnight at room temperature.

The reaction mixture was extracted with ethyl acetate after the addition of 5 mL water. The combined organic phases were washed with 10 % HCl and dried with Na_2_SO_4_. After filtration and concentration 200 mg crude product was obtained. ^1^H NMR (500 MHz, CDCl_3_): *δ*=7.12 (s, 2H, *p*‐cymene), 7.11 (s, 2H, *p*‐cymene), 6.50 (d, *J*=8.5 Hz, 1H), 6.41 (d, *J*=8.5 Hz, 1H), 2.57 (sept, *J*=7.0 Hz, 1H), 2.32 (s, 3H, *p*‐cymene), 2.07–1.97 (m, 2H), 1.93 (sept, *J*=7.0 Hz, 1H,), 1.53–1.50 (m, 2H), 1.38 (s, 3H), 1.24–1.23 (m, 6H, *p*‐cymene), 1.00 (d, *J*=7.0 Hz, 6H).

**Ascaridole synthesis with CiVHPO**: 5 mL 10 mM KBr solution and 5 mL 2 mM NaVO_3_ given were mixed with 90 mL citrate buffer, pH 5, and 10 mL acetonitrile. 0.39 mL α‐terpene (2.4 mmol) were added. The mixture was stirred for 10 min before 2 g urea/H_2_O_2_ (9.2 equiv.) were added or hydrogen peroxide added dropwise. The reaction mixture was stirred overnight at room temperature.

**Optical absorption spectra**: UV/Vis analysis was performed with a Shimadzu spectrometer (1650PC). Spectra were taken immediately after H_2_O_2_ injection. The enzyme concentrations were 15 μM (HRP) and 5 μM (*Mro*UPO).

## Conflict of interest

The authors declare no conflict of interest.

## Supporting information

As a service to our authors and readers, this journal provides supporting information supplied by the authors. Such materials are peer reviewed and may be re‐organized for online delivery, but are not copy‐edited or typeset. Technical support issues arising from supporting information (other than missing files) should be addressed to the authors.

Supplementary
