# Supplementary material for: Singlet‐Oxygen Generation by Peroxidases and Peroxygenases for Chemoenzymatic Synthesis
Source: Chembiochem. 2020 Oct 5;22(2):398–407. doi: 10.1002/cbic.202000326 (PMC7891382; doi:10.1002/cbic.202000326)
Supplement: Supplementary file 1 — Supplementary [file CBIC-22-398-s001.pdf]

ChemBioChem

Supporting Information

Singlet-Oxygen Generation by Peroxidases and Peroxygenases for Chemoenzymatic Synthesis

Kim N. Ingenbosch, Stephan Quint, Melanie Dyllick-Brenzinger, Dennis S. Wunschik, Jan Kiebist, Philipp Süss, Ute Liebelt, Ralf Zuhse, Ulf Menyes, Katrin Scheibner, Christian Mayer, Klaus Opwis, Jochen S. Gutmann, and Kerstin Hoffmann-Jacobsen*

Author Contributions

K.I. Investigation:Lead; Writing - Original Draft:Equal
S.Q. Investigation:Equal; Writing - Original Draft:Supporting
M.D.-B. Investigation:Equal; Project administration:Equal; Visualization:Equal; Writing - Review & Editing:Equal
D.W. Validation:Equal; Visualization:Equal; Writing - Original Draft:Supporting
J.K. Investigation:Supporting; Resources:Equal; Writing - Review & Editing:Supporting
P.S. Validation:Supporting; Writing - Review & Editing:Supporting
U.L. Project administration:Equal; Validation:Equal
R.Z. Conceptualization:Equal; Funding acquisition:Equal; Supervision:Equal
U.M. Conceptualization:Equal; Funding acquisition:Equal
K.S. Resources:Equal; Validation:Supporting; Writing - Review & Editing:Supporting
C.M. Supervision:Equal; Writing - Review & Editing:Supporting
K.O. Conceptualization:Equal; Funding acquisition:Equal; Project administration:Equal; Validation:Equal
J.G. Resources:Equal; Supervision:Equal
K.H.-J. Conceptualization:Equal; Formal analysis:Lead; Supervision:Equal; Writing - Original Draft:Equal

Supporting Information

1. Analysis of singlet oxygen produced by photochemical synthesis with Rose Bengal

Singlet oxygen was produced by irradiation of a 7.33 µM solution of rose Bengal in phosphate buffer with a cw 543 nm diode laser (REO 30967, Research Electro-Optics, Inc., Boulder, USA) at a laser power of 0.5 mW. SOSG was applied in the same concentration as in the enzyme experiments, i.e. 0.55 µM. A linear curve was obtained for the entire range of the fluorescence intensity as a function of the irradiation time. This confirms that the intensity is directly proportional to the singlet oxygen concentration in all experiments performed in this work.

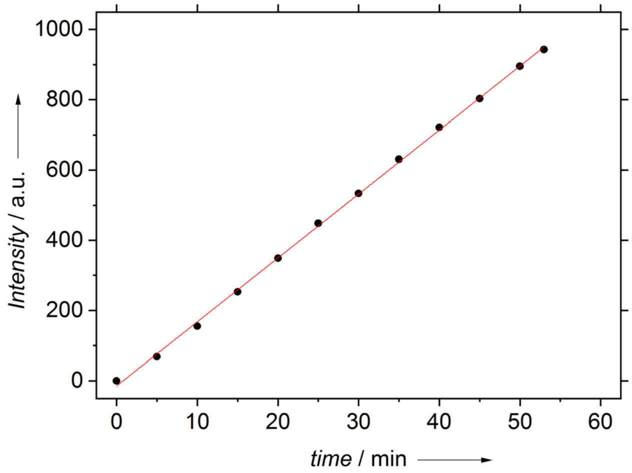

Figure S1: Dependence of the fluorescence intensity on the irradiation time of a Rose Bengal solution producing singlet oxygen. Singlet oxygen is trapped with SOSG as the fluorescent SOSG-EP.

2. Effect of SOSG concentration on fluorescence intensity

In order to investigate whether the fluorescence intensity is limited by the SOSG concentration, hydrogen peroxide and SOSG were used stoichometrically in the concentration range from 1 to 10 µM. Here, a quantitative spectroscopic analysis is feasible. In contrast to Figure 3 of the main article an increasing amount of $^1O_2$ can be detected by SOSG-EP with increasing hydrogen peroxide concentration. (Figure S2). This shows that the plateau observed for peroxidases in the µM to mM concentration range is an artifact due to the lack of sufficient SOSG for complete $^1O_2$ capturing. It must be noted that this analysis requires a reduction of the detector voltage so that

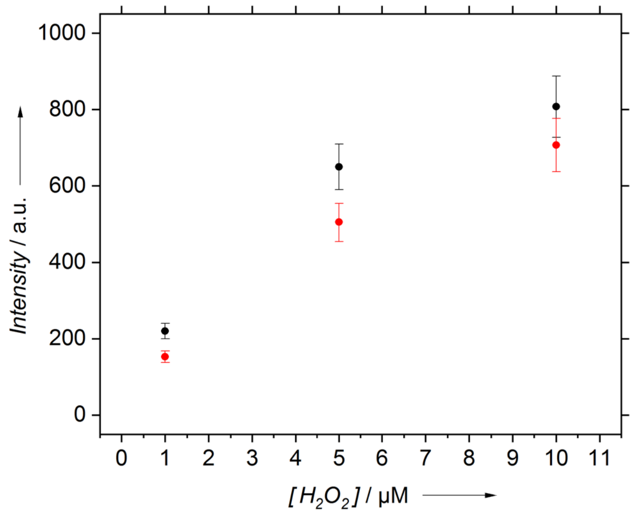

Figure S2: SOSG-EP intensity observed with MaxiBright (red) and HRP (black) at different H_2O_2 concentrations when the SOSG and H_2O_2 are supplied in equimolar amounts.

the intensities of Figure 3 and Figure S2 are not quantitatively comparable. HRP shows higher SOSG-EP intensities as MaxiBright revealing higher singlet oxygen yields. Yet, these intensity differences are more pronounced under SOSG scarcity. This underlines the possibility of side reactions if singlet oxygen is not completely quenched, which is more pronounced in the case of the enzyme giving highest reaction rates.

3. Kinetics of enzymatic singlet oxygen generation and subsequent trapping of singlet oxygen with SOSG

The kinetic equations for the mechanism illustrated in Fig. 4 are set up and solved with the steady state approximation. As singlet oxygen is a reactive intermediate the transient concentration is approximately zero. Michaelis Menten kinetics were assumed for the enzymatic formation of singlet oxygen. Under these assumptions the rate law for singlet oxygen formation is given by

$$\frac{d[\,^1O_2]}{dt} = \frac{k_{cat} \cdot [E] \cdot [S]}{K_M + [S]} - k_\Delta \cdot [\,^1O_2] - k_2[\,^1O_2] \cdot [SOSG] = 0 \qquad \text{(Eq. S1)}$$

with E depicting the enzyme, S the substrate H_2O_2 and K_M the Michaelis constant for substrate binding.

The equation can be solved for the singlet oxygen concentration.

$$[\,^1O_2] = \frac{k_{cat} \cdot [E] \cdot [S]}{K_M + [S]} \cdot \frac{1}{k_\Delta + k_2 \cdot [SOSG]} \qquad \text{(Eq. S2)}$$

The formation of the fluorescent SOSG endoperoxide is assumed to be a second order reaction.

$$\frac{d[SOSG - EP]}{dt} = k_2 \cdot [SOSG] \cdot [\,^1O_2] \qquad \text{(Eq. S3)}$$

Substituting the concentration of singlet oxygen in Eq. S3 by Eq. S2 gives

$$\frac{d[SOSG - EP]}{dt} = \frac{k_2 \cdot [SOSG]}{k_\Delta + k_2 \cdot [SOSG]} \cdot \frac{k_{cat} \cdot [E] \cdot [S]}{K_M + [S]} \qquad \text{(Eq. S4)}$$

This is Eq. 2 given in the main article.

4. NMR analysis of the raw product of ascaridole synthesis

The NMR of the crude product is shown in Figure S3. Besides the signals of the double bond a singlet for the methyl group of ascaridole (1.38 ppm) can be detected. The ascaridole content of the crude product was determined using the region where the signals for the methyl groups of the isopropyl group appear (0.8-1.05 ppm) as reference to the characteristic ascaridole signals.

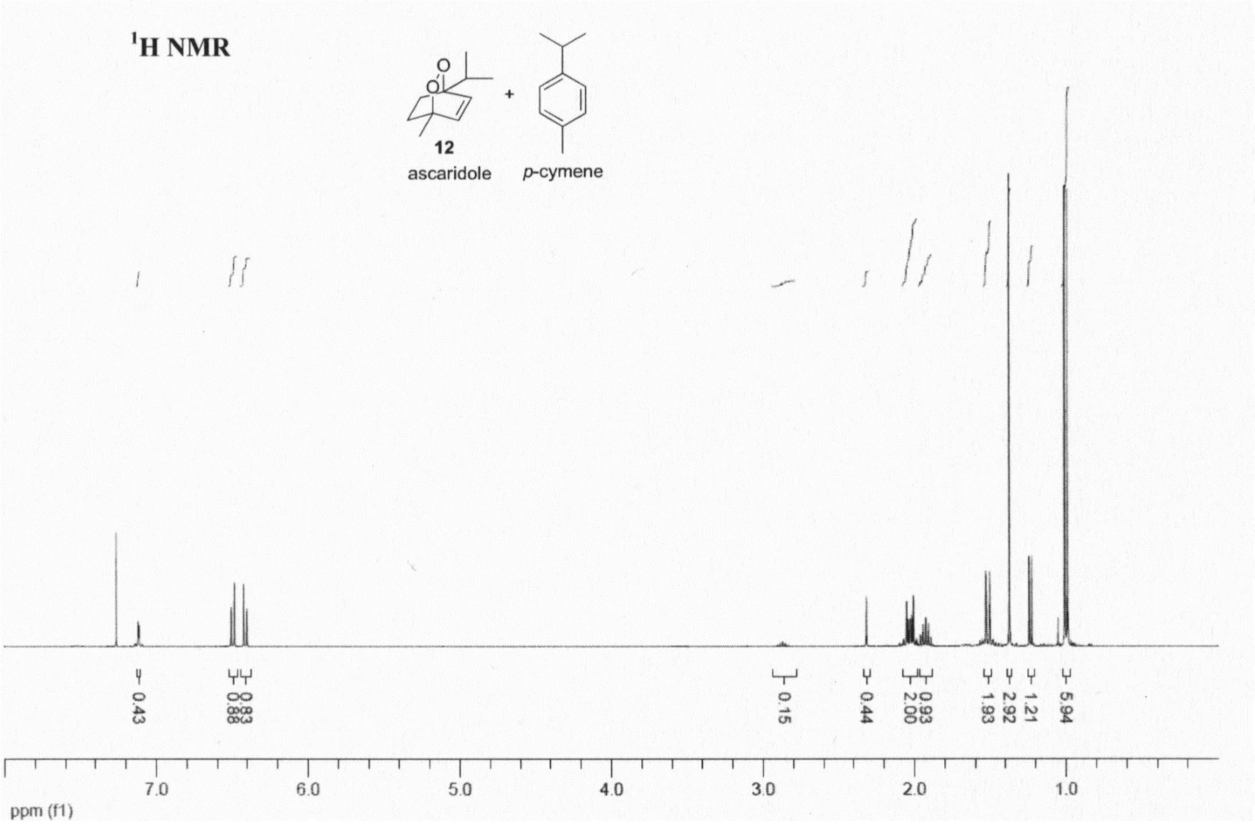

Figure S3: ^{1}H NMR (500 MHz, $CDCl_3$) spectrum of the crude product.

5. Absorption spectra of the peroxidases and peroxygenases

Absorption spectra in the high hydrogen peroxide concentration regimes were acquired in 1 mM hydrogen peroxide with HRP and in 2.5 mM hydrogen peroxide with *Mro*UPO. Whereas further bleaching is observed with *Mro*UPO, HRP shows absorption maxima at 587 nm and 544 nm which indicate compound III formation (Figure S4). The last sampling time reveals the formation of the inhibited verdohaemochrome species P-670.

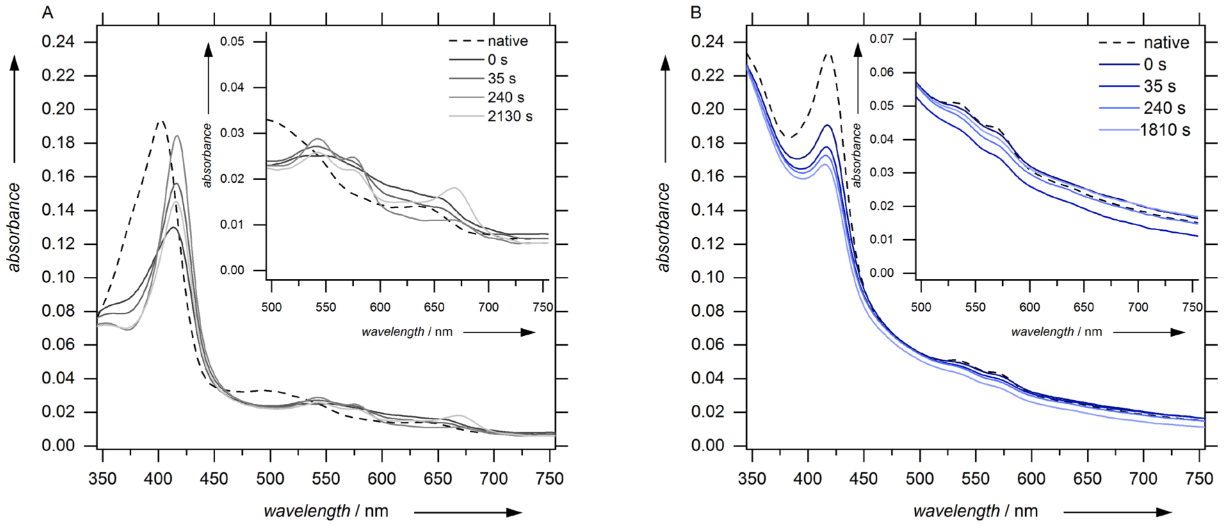

Figure S4: Optical absorption spectra of HRP (left) and *Mro*UPO (right) at high H_2O_2 concentrations (HRP: 1 mM, MroUPO: 2.5 mM).

The absorption spectra of HRP in the presence and absence of two mass equivalents of SOD is shown in Figure S5. No major differences are observed apart from the kinetic inhibition by SOD.

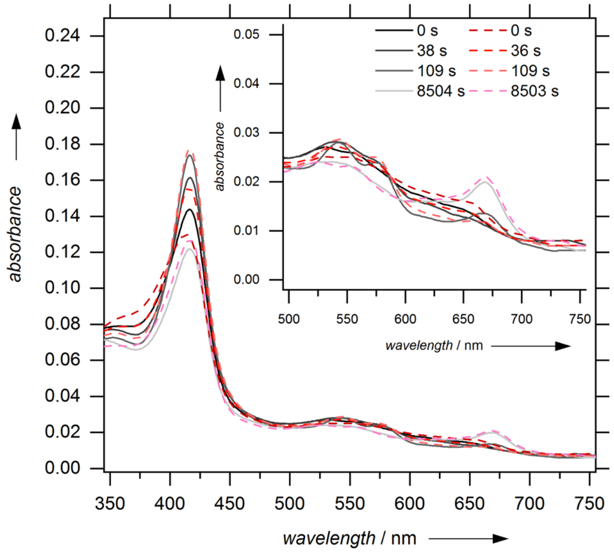

Figure S5: Absorption spectra of HRP in 1 mM H_2O_2 in the presence (grey) and absence (red) of two mass equivalents of SOD.
